# UCHL1 expression and localization on testicular development and spermatogenesis of Chinese giant salamanders

**DOI:** 10.18632/oncotarget.20910

**Published:** 2017-09-15

**Authors:** Yuanxian Wang, Liqing Wang, Huihui Gao, Yao Gao, Changming Yang, Hong Ji, Wuzi Dong

**Affiliations:** ^1^ College of Animal Science and Technology, Northwest A&F University, Yangling 712100, China; ^2^ Animal Husbandry and Veterinary Station of Chenggu County, Hanzhong 723200, China

**Keywords:** Chinese giant salamander, UCHL1, male gonad development, extracellular vesicles, germ cell niches

## Abstract

Ubiquitin carboxyl-terminal hydrolase L1 (UCHL1), which is extensively expressed in vertebrates, is a deubiquitinating enzymes that inhibits the degradation of proteins by reversing ubiquitination modification. Herein, a 1087-bp sequence encoding UCHL1 was identified from the Chinese giant salamander (CGS; *Andrias davidianus*). The coding sequences (CDS) of *UCHL1* encoded a putative poly peptide of 222 amino acids. The CGS UCHL1 isoforms were more related to their human and mouse counterparts. The phylogenic tree of vertebrate UCHL1 indicated that CGS UCHL1 has the closest relationship with human UCHL1 (up to 73.99 %). Before the gonads of male CGSs matured, the peak level of UCHL1 expression in testes appeared in 3-year-old CGSs according to RT-qPCR and western blot. In adult testes, the level of UCHL1 protein was lower in the breeding period than in the post-breeding period, whereas the level of UCHL1 protein in interstitial fluid of adult CGS testes was higher during the breeding period than during the post-breeding period. In testicular seminiferous lobules in the developmental stage of CGSs, immunohistochemistry displayed three kinds of localizing patterns of UCHL1, including nuclear localization at half year old, cytoplasmic localization from one year to three years old, and extracellular localization in adult. In testicular seminiferous lobules of adult CGS, the different developmental germ cells were separated by cysts containing UCHL1 protein, but UCHL1 did not localize on the mature sperm. The results showed that extracellular UCHL1 loaded on exosomes, as a component of the homogeneous germ cell cysts, could regulate the synchronous development of sperm in testes of adult CGS.

## INTRODUCTION

Ubiquitination modification is a ubiquitous modifying method of post-translation of proteins to induce protein degradation, which can be regulated and reversed by deubiquitinating enzymes [1, 2]. UCHL1 also known as PGP9.5, is a deubiquitinating enzyme that hydrolyzes C-terminal esters and amides for generating monomeric ubiquitin [3]. UCHL1 appears to be a general marker of nerves and neuroendocrine cells across the vertebrate kingdom [3], i.e., mammals, birds, reptiles, amphibians, fish, and even marsupials. UCHL1/PGP 9.5 has also been reported in various locations in numerous species, including rainbow trout, *Oncorhychus mykiss* [4]; barfin flounder, *Verasper moseri* [5]; penguins, *Spheniscidae* Family [6]; Surinam caiman, *Caiman crocodilus crocodilus* [7]; flying fox, *Pteropus vampyrus* [8]. This pattern suggests that UCHL1 is a conservative protein enzyme involved in special biological functions.

Different localization of UCHL1 plays an important role in regulating cellular proliferation and differentiation by ubiquitination modification of post-translational proteins. Three existence forms of UCHL1 appear in animals, including cytoplasmic localizing, nuclear localizing, and secretory UCHL1. Cytoplasmic localization of UCHL1 is the main localization in cells that serve as markers for the non-proliferating spermatogonia in primates [9], and neurons in mice and humans. Nuclear localization of UCHL1 occurs in approximately 5% of neurons in the central and peripheral nervous systems [10], but the function is unknown. Extracellular UCHL1 in the body fluid and spaces within tissues has also been reported. UCHL1/PGP 9.5 in serum is measured at approximately 100 ng/ml [10]. There have been several reports regarding UCHL1/PGP 9.5 localization in the renal tubules of mice and its distribution in the epididymis of rat [11], human [12], and mouse [13]. It was thought that the expression of UCHL1/PGP 9.5 in any part of the renal tubule or the epididymal cauda was possibly caused by re-absorption from plasma [11] or circulatory system, because there was no expression of UCHL1 mRNA in the renal tubule or in the epididymal cauda [10].

The ubiquitin pathway plays a critical role in the progression of spermatogenesis through the mitotic, meiotic, and post-meiotic phases [14]. Generally, conserved expression of UCHL1 regulates spermatogensis in mammalian testes [15]. UCHL1 is expressed in spermatogonia and Sertoli cells in rodents [16], whereas in other mammals, including humans, non-human primates [14], and domestic animals, such as bovines [17, 18], pigs [19], goats [20, 21], it is only expressed in gonocytes and spermatogonia in seminiferous tubules. UCHL1 is considered an optimal marker for spermatogonia and for pre-meiotic male germ cells [16]. For example, in fish, UCHL1, via the ubiquitin-proteasome system, may play an important role in the gonadal transformation process in the rice field eel (*Monopterus albus*) [22]. In amphibians, UCHL1 is possibly involved in the process of progesterone-induced toad oocyte maturation [23].

The Chinese giant salamander (CGS, *Andrias davidianus*) is the largest extant amphibian in the world [24, 25], belonging to the *Cryptobranchidae* family, which only contains three species: (*Cryptobranchus alleganiensisin* in North America, *Andrias japonicus* in Japan, and *Andrias davidianus* in China). As an endangered species, the CGS has received increasing attention in evolutionary, comparative biology, and other studies [26-29]. Although there are morphological differences between urodele testis and mammal testis, the spermatogenetic process in urodele amphibians is similar to that of mammals. Many genes take part in regulating the process of spermatogenesis. To date, the molecular characterization and cellular localization of UCHL1 has not been reported during development of the CGS gonad. In addition, the potential function of UCHL1 in spermatogenesis of CGS is not clear. In this study, the cDNA sequence of UCHL1 was identified and characterized for the Chinese giant salamander and the potential function of UCHL1 was analyzed during spermatogenesis and the development of CGS gonads.

## RESULTS

### Sequence characterization of UCHL1

**Figure 1:**
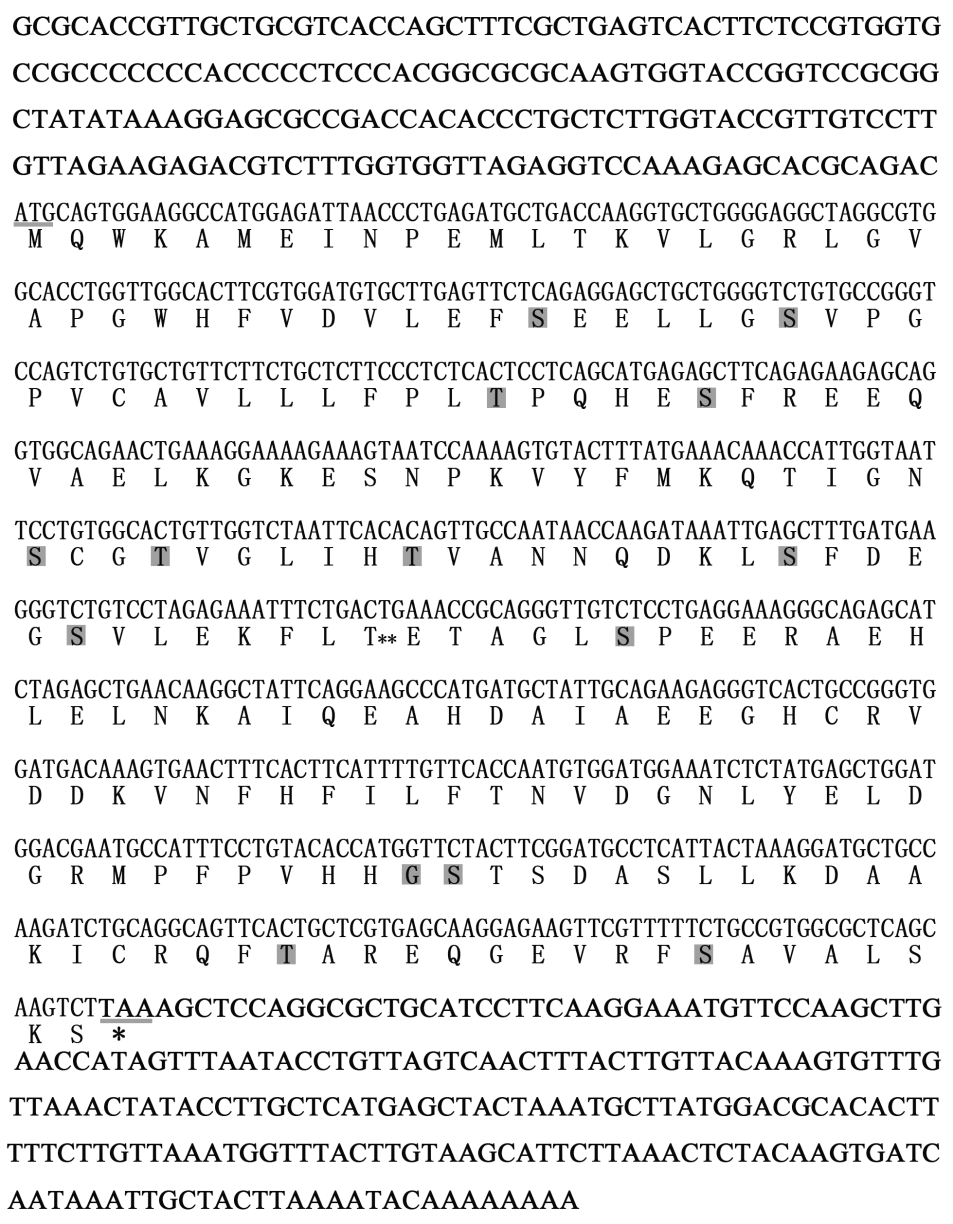
Characterization of amino acid sequences and cDNA nucleotide sequences of CGS UCHL1 The start codon and stop codon are underlined. The gray boxes show the phosphorylation sites. The double asterisks (**) display the O-linked glycosylation site.

A fragment of UCHL1 was obtained from the transcriptome database for the Chinese giant salamander. It was confirmed by specific amplification with primers UCHL1-F and UCHL1-R. The total sequence of UCHL1 mRNA has been obtained by 3′ and 5′ RACE and deposited in the GenBank database under accession number (MF418646). The cDNA sequence consisted of 1087 bp, including a 195 bp 5′ UTR, 223 bp 3′ UTR, and a 669 bp ORF with content of G + C (48.6 %) (Figure 1). The CDS of UCHL1 encoded a putative polypeptide of 222 amino acids with a calculated molecular mass of 24.6 kDa and a theoretical pI of 5.19. SignalP analysis showed the typical signal peptide was not found. The number of the potential phosphorylation sites and O-linked glycosylation sites were 14 and one, respectively (Figure 1).

**Figure 2:**
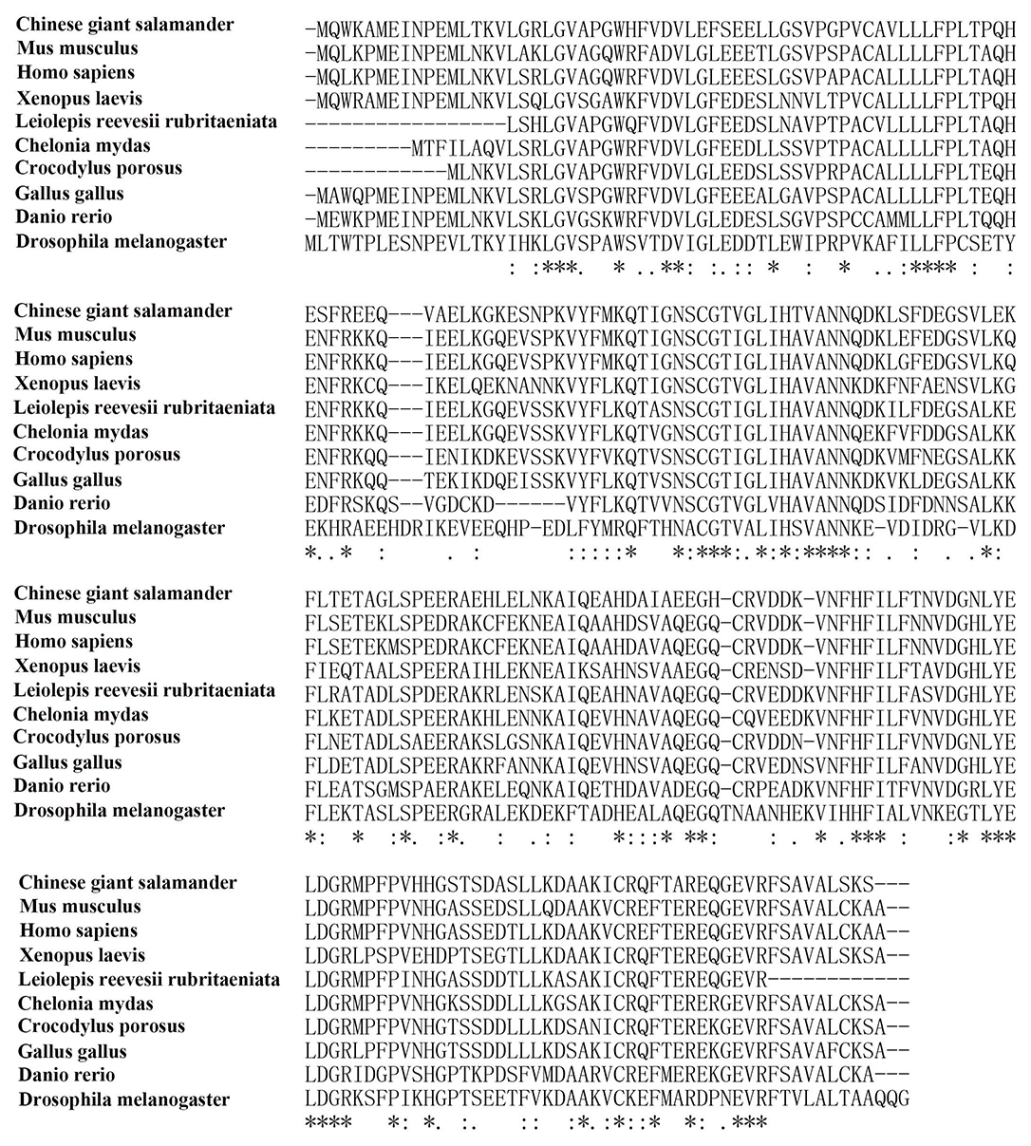
Multiple alignments of UCHL1 amino acid sequences of 10 species

**Figure 3:**
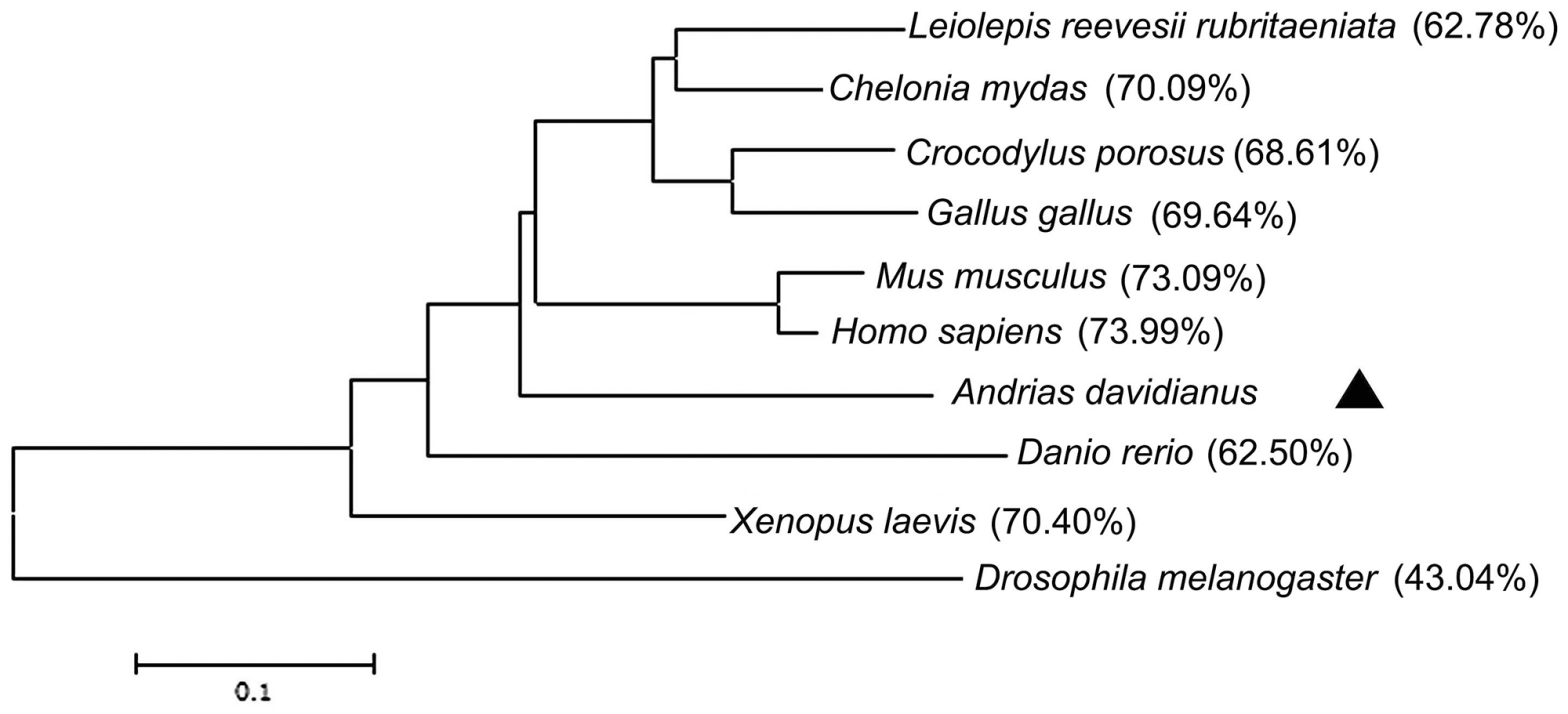
Phylogenetic tree of UCHL1 and other homologs The phylogenetic tree was constructed based on the full-length amino acid sequences by the neighbor-joining method and the bootstrap value was set at 1000. The CGS UCHL1 is labeled with triangle (▲). Percentage of bootstrap replications is shown in the figure. The scale bar (0.01) represents the genetic distance.

The results of Blast showed that amino acid sequences of CGS UCHL1 was most homologous with human UCHL1 (up to 73.99 %), secondly that of mice (up to 73.09 %) and then *Xenopus. laevis* (up to 70.4 %) (Figure 2). The phylogenetic analysis demonstrated that the phylogeny of CGS UCHL1 and human UCHL1 was more closely related than that of any other vertebrate (Figure 3).

### Expression level of UCHL1 mRNA in testes of CGSs at different developmental stages

**Figure 4:**
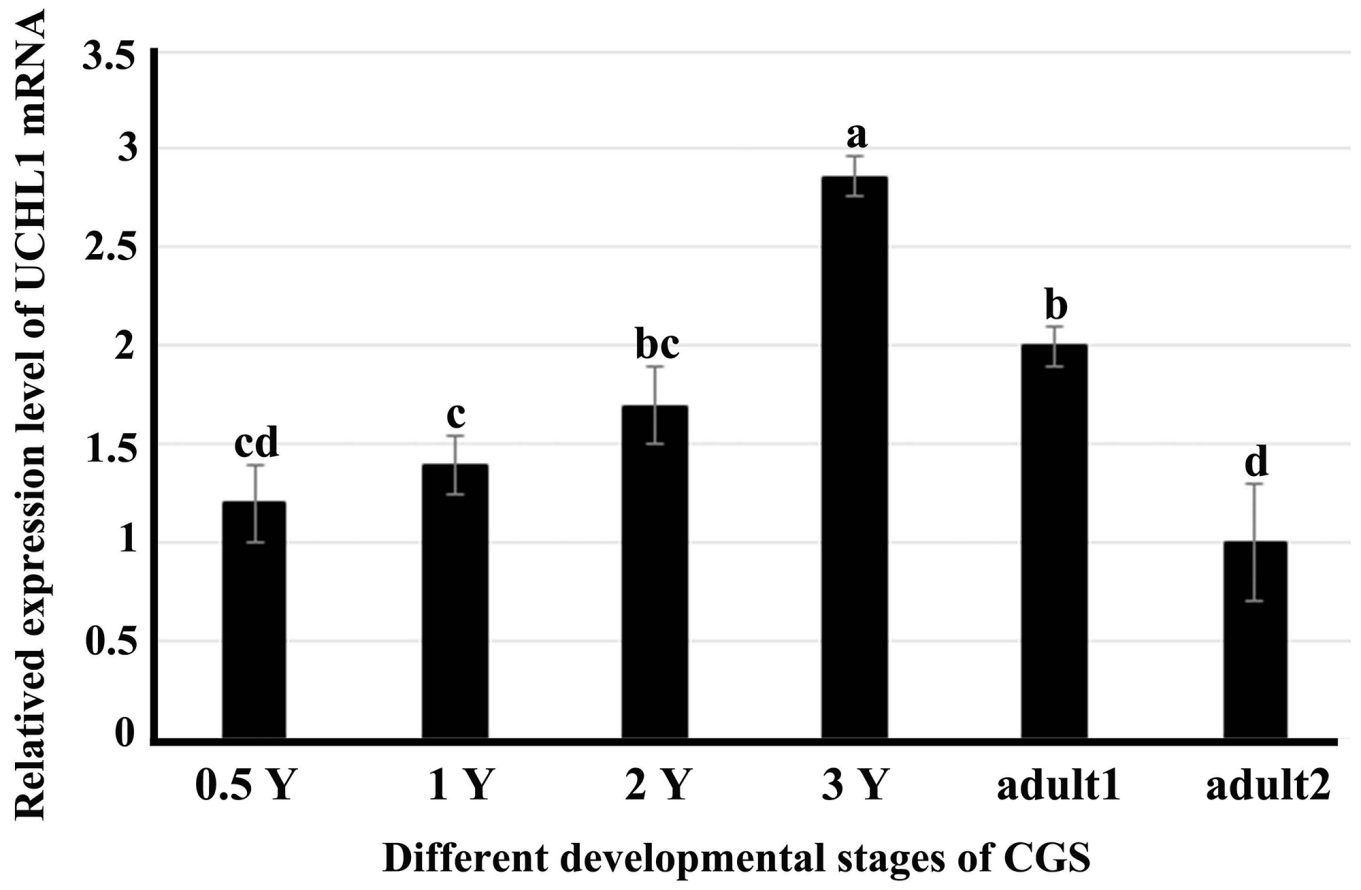
Expression profiles of UCHL1 mRNA in male gonads at different developmental stages by real-time PCR with β-actin as an internal reference 0.5 Y, 1 Y, 2 Y, and 3 Y represented samples of testes or gonads from 0.5-year-old, 1-year-old, 2-year-old, 3-year-old and adult CGSs. Adult1 denoted the tissue samples of adult CGSs in November (post-breeding period); adult2 denoted the tissue samples of adult CGSs in August (breeding period). Similar letters denote non-significant differences (*p* > 0.05). Different letters indicate a significant difference (*p* < 0.05).

The RT-qPCR was employed to examine the level of UCHL1 in testes of CGSs at different developmental stages (Figure 4). We found that UCHL1 mRNA was expressed in all different developmental testis tissues. The peak level of UCHL1 mRNA appeared in testes of 2-year-old CGSs. Further, the expression level of UCHL1 in adult testes (at least 4 years old) was lower during the breeding period (August) than in the post-breeding period (November).

### Localization of UCHL1 in testis of CGSs at different developmental stages by IHC

As stated above, the amino acids sequences of UCHL1 protein were highly conservative. An antibody of human UCHL1 was used to localize UCHL1 in the testes of CGSs at different developmental stages by IHC.

**Figure 5:**
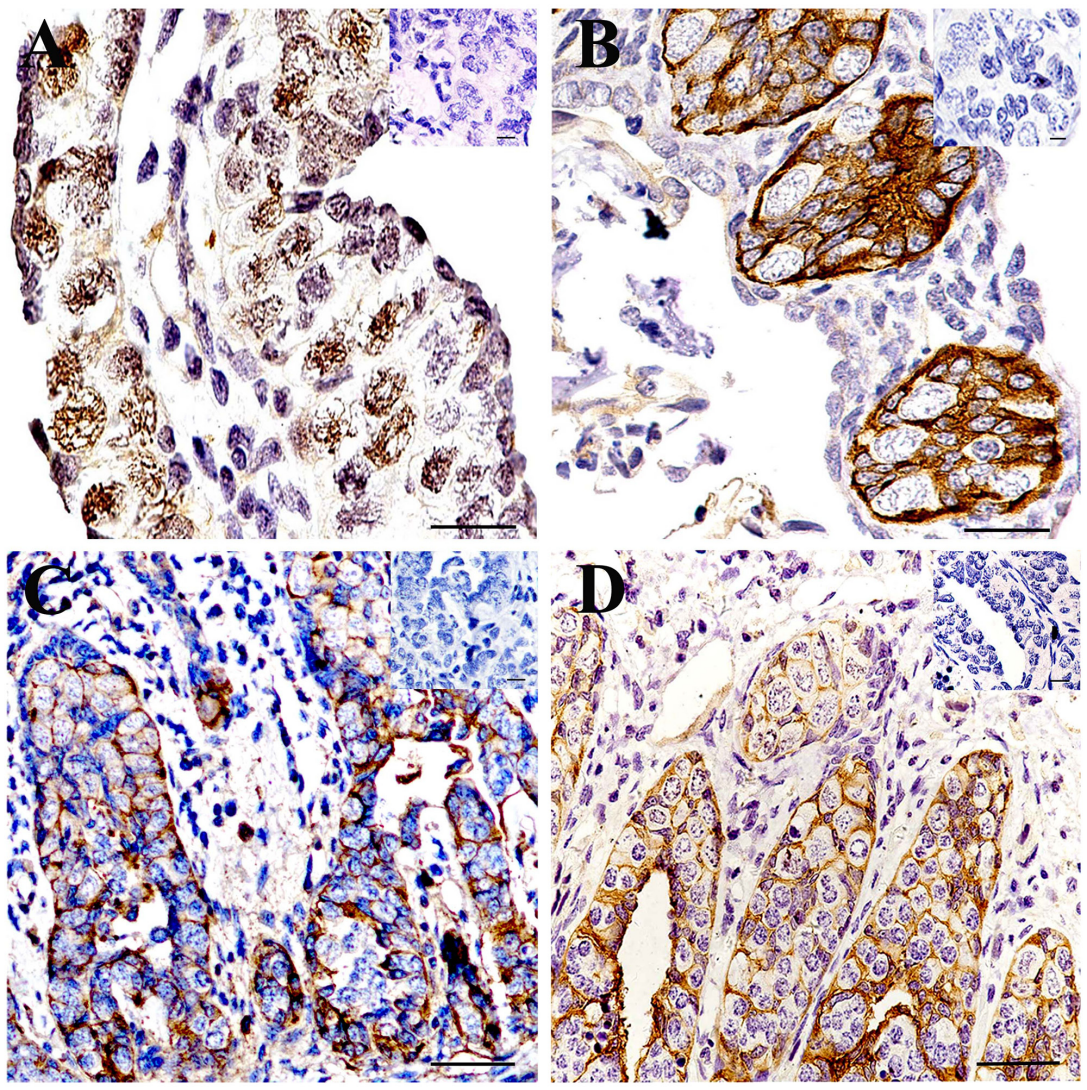
Localization of UCHL1 in gonads at different developmental CGS by IHC **(A)** 0.5-year-old CGSs; **(B)** 1-year-old CGS; **(C)** 2-year-old CGS; **(D)** 3-year-old CGS. The inset represents the negative controls. Scale bar, 80 μm.

In the gonad of the 0.5-year-old CGSs, UCHL1 was located in the nucleus of large cells with loose chromatin, which was distributed in the peripheral region of the gonads (Figure 5A).

In the testes of the 1-year-old CGSs, these UCHL1 positive cells aggregated together to form the primary seminiferous lobules without a lumen. UCHL1 was only distributed on the membrane of the cells in seminiferous lobules (Figure 5B).

In histologically, the lumen of seminiferous lobules of CGSs had formed after 2 years of age (Figure 5C) and the lumen was smaller than those of 3-year-old CGSs (Figure 5D). The UCHL1 positive cells displayed a tight junction state to form the lobular lumen wall at this stage (Figure 5C, 5D). In the testes of the 3-year-old CGSs, the UCHL1 positive cells were regularly arranged in the seminiferous lobules wall (Figure 5D).

**Figure 6:**
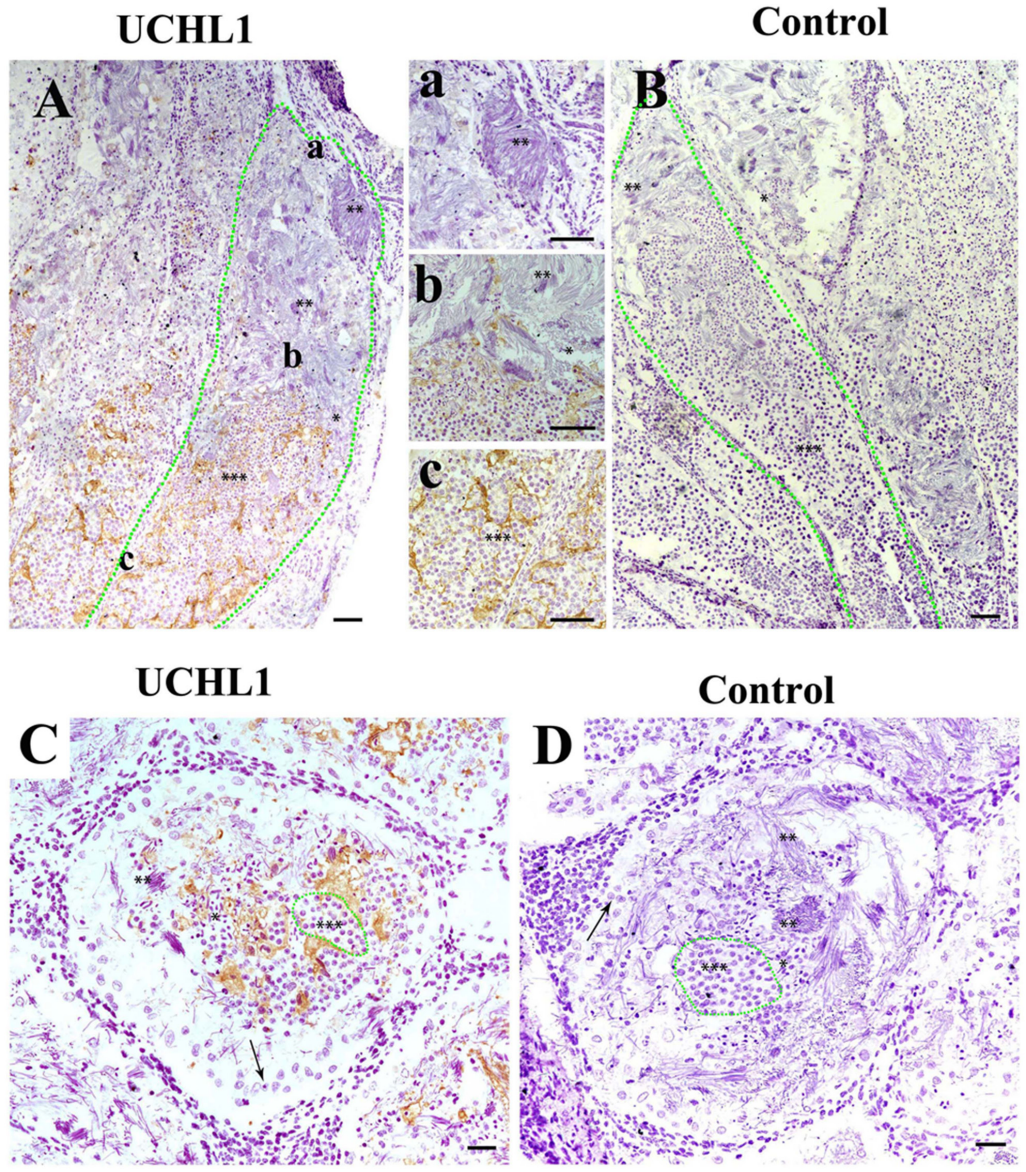
Localization of UCHL1 in male gonads of adult CGS by IHC The different morphological sperm was in seminiferous lobules, i.e., the round spermatid (***), sperm with condensed nucleus (**); sperm with non-condensing nucleus (*). **(A, B)** Vertical section. The zone of green dots denotes a seminiferous lobule. a, b, and c represent the enlarged corresponding regions of Figure A, respectively. **(C, D)** Transverse section. The zone of green dots denote a speculative cyst. Arrows denote a type of large cell near the well of seminiferous lobules. Scale bar, 200 μm.

In testes of adult CGSs, the expression of UCHL1 significantly decreased in the different segments of the seminiferous lobules. From the proximal to the distal end of the seminiferous lobules, the expression level of UCHL1 gradually decreased, until it was no longer detected (Figure 6A). Interestingly, UCHL1 was distributed in the extracellular matrix, and little of the UCHL1 positive signal appeared in the cells of the seminiferous lobules (Figure 6). The seminiferous lobule was divided into different cysts by the extracellular UCHL1. High levels of UCHL1 expression occurred in the proximal seminiferous lobule where there were germ cells at different developmental stages. Meanwhile, no UCHL1 expression occurred in the distal end of the seminiferous lobule, which was full of mature sperm (Figure 6).

### Developmental expression of UCHL1 protein in testes of CGSs at different stages

**Figure 7:**
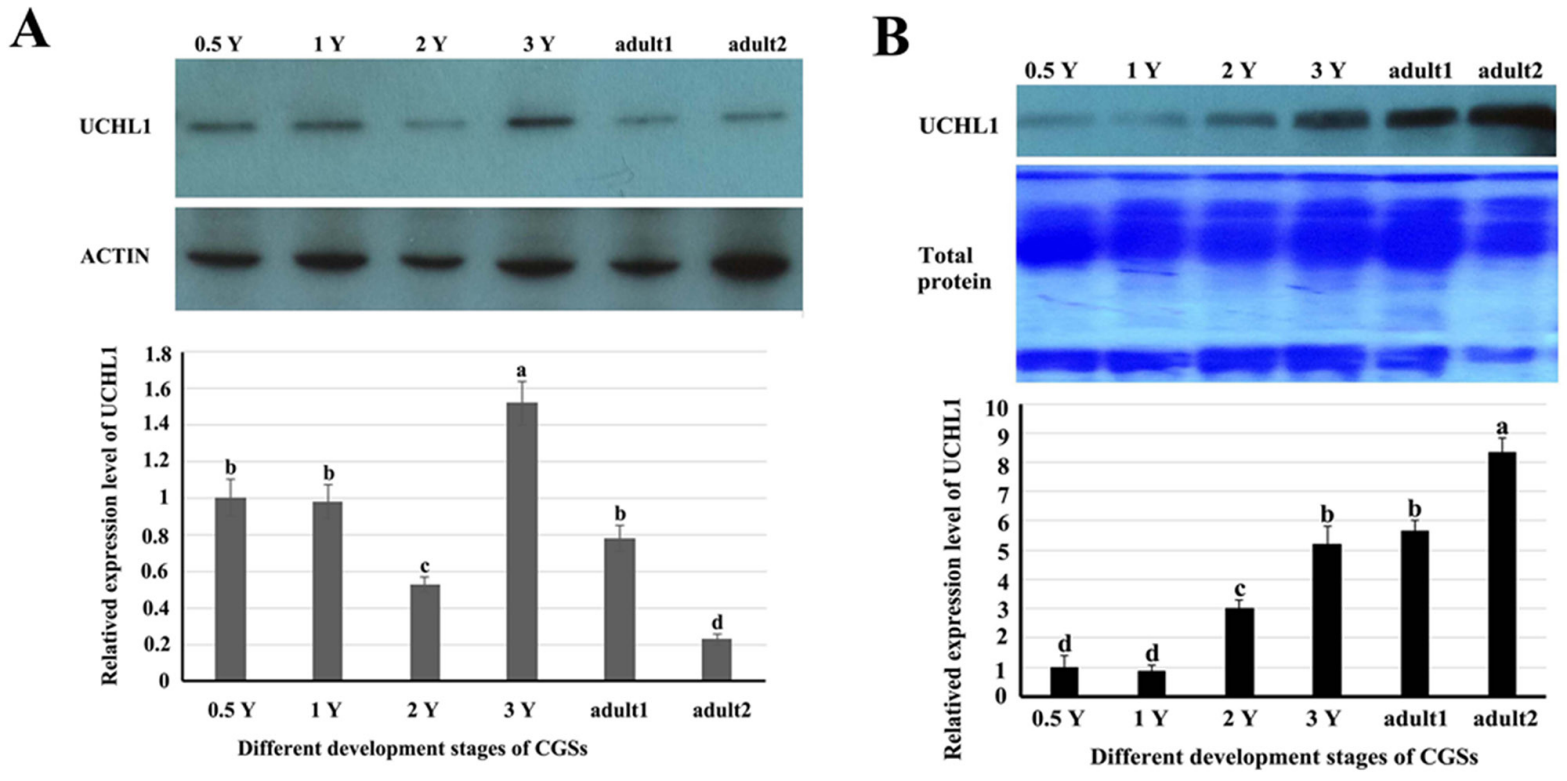
The related expression level of UCHL1 protein in the tissues or in the leaking liquid of male gonads at different developmental stages **(A)** The level of UCHL1 protein in the tissues of male gonads at different developmental stages. 0.5 Y, 1 Y, 2 Y, 3 Y, adult1, and adult2 represented samples of gonads from 0.5-year-old, 1-year-old, 2-year-old, 3-year-old, adult CGSs in November, and adult CGSs in August, respectively. **(B)** The level of UCHL1 protein in the tissues or in the leaking liquid of male gonads at different development stages. 0.5 Y, 1 Y, 2 Y, 3 Y, adult 1, and adult2 represent the samples of the gonads leaking liquid from 0.5-year-old, 1-year-old, 2-year-old, 3-year-old, adult CGSs in November, and adult CGSs in August, respectively. Same letters denote non-significant difference (*p* > 0.05). Different letters indicate a significant difference (*p* < 0.05).

**Figure 8:**
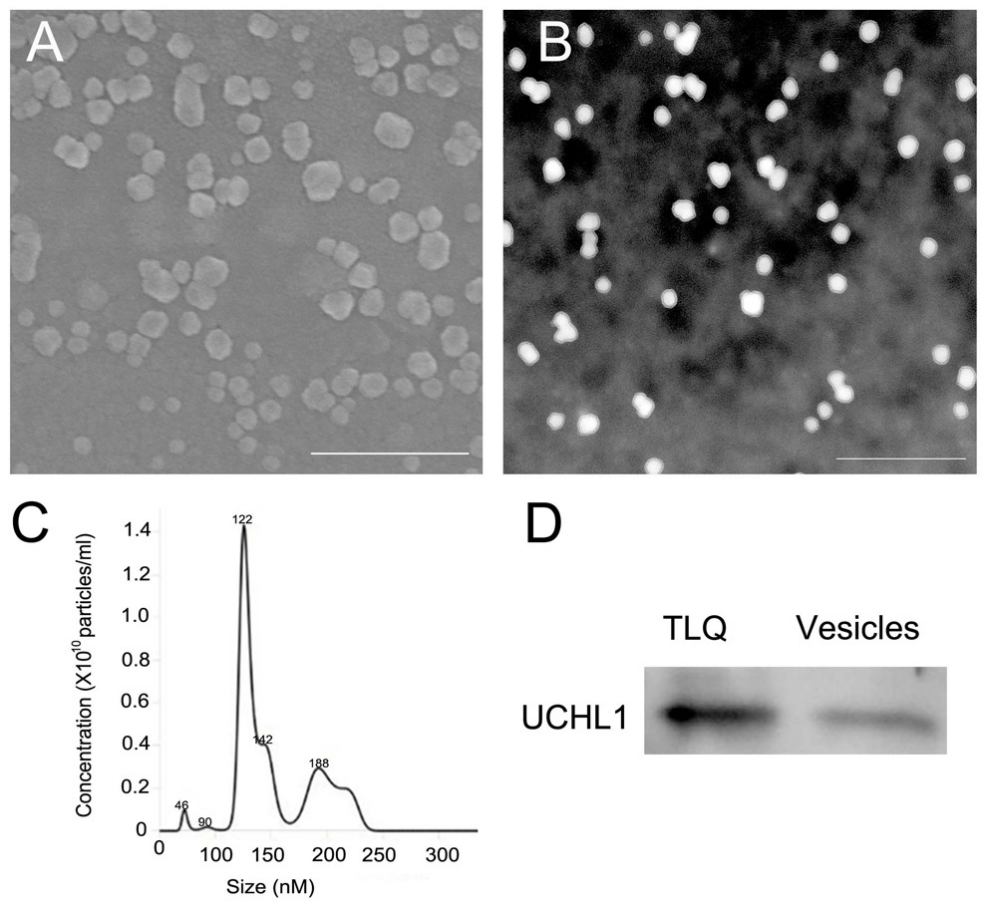
Detection of CGS UCHL1 in extracellular vesicles from the leaching liquid of the adult testes **(A)** Detection of the vesicles by scanning electron microscopy. **(B)** Detection of the vesicles by transmission electron microscopy. **(C)** Size distribution and quantification of the extracellular vesicles analyzed by NanoSight NS300. **(D)** Detection of UCHL1 protein by western blot. TLQ represented the protein samples from tissue leaching liquid of adult testes; vesicles represent the protein samples from extracellular vesicles of the tissue leaching liquid. Scale bar: 500 nm.

Western blot was performed to detect UCHL1 protein level of testes at different developmental stages (Figure 7). The testis tissue was divided into the pellets and the supernatant fractions. The levels of UCHL1 in testicular tissue pellets removed and tissue fluid are shown in Figure 7A; furthermore, the expression level of UCHL1 protein in testicular leaching liquid is displayed in Figure 7B (see materials and methods). The peak level of UCHL1 protein in testis pellets appeared for the 3-year-old CGSs, however, the level of UCHL1 protein was lowest in testes of the 2-year-old CGSs. In adult testes, the expression level of UCHL1 protein was lower in the breeding season (August) than in the post-reproduction period (November) (Figure 7A). Furthermore, the level of UCHL1 protein in testicular leaching liquid was gradually increased with male gonad development. Interestingly, the expression level of UCHL1 protein in testicular leaching liquid was higher in breeding period than after reproduction in adult testes (Figure 7B). Furthermore, UCHL1 protein was detected in the exosomes from testicular leaching liquid (Figure 8).

## DISCUSSION

UCHL1 (or PGP 9.5) is a conservative protein extensively presents in animals, including nematodes, Drosophila, fish, amphibians, birds, and mammals [3]. In this study, the sequence of CGS UCHL1 cDNA was obtained and the molecular characteristics were revealed by bioinformation analysis. CGS UCHL1 was most homologous with human UCHL1 (up to 73.99 %) among 10 different species compared. In the phylogenetic tree, CGS UCHL1 was clustered together with the UCHL1 of other higher vertebrates. This illustrated that the evolutional characterization of CGS UCHL1 is conservative. The homology of CGS UCHL1 and *X. laevis* UCHL1 was also very high (up to 70.04 %), whereas *X. laevis* UCHL1 formed a clade in the phylogenetic tree with CGS and other vertebrates (Figure 3). It was inferred that CGS UCHL1 could play a special function in CGSs.

UCHL1 was initially found to be expressed specifically in the human brain [33]. The mRNA for UCHL1/PGP 9.5 is known to be expressed extremely early in the differentiation of the nervous system [34]. Subsequent research identified UCHL1 was prominently present in the kidneys [35], ovaries [36], and testes [19, 21]. In mammals, UCHL1/PGP 9.5, expression in spermatogonia cells, was found to play an important role in spermatogenesis [9, 19]. In this study, we observed regular expression of UCHL1 during the gonad development of the Chinese giant salamander. The peak level of UCHL1 mRNA appeared in testes of 3-year-old CGSs, as well as the expression pattern of UCHL1 protein in testis tissues (Figure 4, 6A). Specially, the expression level of UCHL1 mRNA and protein in adult testes was lower during the breeding season than during the post-reproduction period (Figure 7A). However, the expression level of UCHL1 protein in the testicular leaking liquid of adult CGSs was higher during the breeding season than during the post-reproduction period (Figure 7B). The results suggested that UCHL1 played an important role in regulating the gonad development of CGSs and the testicular annual cycles.

Three localizations of UCHL1 in tissue have been previously reported [10, 11, 35]. The intracellular and extracellular localization of UCHL1 were also observed in testes during the developmental stages of CGSs. In the seminiferous lobules of CGS testes before sexual maturation, the UCHL1 was only localized on primordial germ cells and Sertoli cells (Figure 5). This proved that UCHL1 could play an important role in regulating the gonadal development of CGSs.

CGS UCHL1 should be thought of as a non-secretary protein because the typical signal peptide in the UCHL1 protein molecular was not found. Although our results showed that the UCHL1 was in the extracellular matrix and loaded in exosomes (Figure 8). The UCHL1 could be partly released into the extracellular matrix through the exosome pathway. In mammals, UCHL1 protein can be transferred by loading on exosomes [37, 38]. The UCHL1 loading on exosomes could have important biological functions in the male gonads of adult CGSs.

In fish and amphibians, there are cysts that could be related with homogeneous development of germ cells in seminiferous lobules after sexual maturity [39, 40]. The exosomes loading UCHL1 could be involved in forming the cysts (or niches) in which the homogeneous germ cells aggregated. The cysts separated germ cells at different stages from each other to ensure the synchronous maturation of sperm in fish and amphibians [41, 42]. The synchronous maturation of sperm allow for the ejection of a copious amount of sperm to fertilize eggs *in vitro* in water during the breeding season of amphibians and fish [43, 44]. It was inferred that the extracellular UCHL1 could construct a barrier to protect normal development of the germ cells by reversing the protein ubiquitination pathway. UCHL1 could also regulate spermatogenesis of adult CGS during the annual testicular cycle.

## CONCLUSION

In conclusion, we have identified UCHL1 from CGS and investigated the expression level and localization pattern of UCHL1 in seminiferous lobules during development of testes. Moreover, during the annual cycle of testicular development in adult CGS, the extracellular UCHL1 in exosomes is an important component of cysts (or niches), which could be related to the synchronous development of germ cells. The present study enriched our knowledge of UCHL1 and provided valuable information for better understanding of the spermatogenesis characteristics of CGS. In the future, the mechanism of UCHL1 regulation in spermatogenesis will be further studied to reveal the reproductive characteristics of CGS.

## MATERIALS AND METHODS

### Collection of tissues samples of Chinese giant salamanders

Chinese giant salamanders were collected from eight farms in Hanzhong County (Shaanxi Province, China) in August and October, 2016. CGSs were anesthetized using 0.6 mg/L MS-222 and sacrificed by destroying the spinal cord of the CGS with a needle. These CGSs were the second or third generation of individuals that were permitted to be used in this study by the Wildlife Conservation Bureau of Shaanxi Province, China. The tissue samples were collected immediately after dissection. Some samples were fixed in Bouin’s liquid for section, and other aliquots were stored in liquid nitrogen for protein and RNA extraction. This study was approved by the Animal Care and Use Committee of Northwest A&F University in China. All protocols were carried out in accordance with the approved guidelines and regulations.

### Total RNA extraction and cDNA synthesis

Total RNA was extracted using RNAiso Reagent following the manufacturer’s protocol. The concentration, purity, and integrity of RNA were detected by the NanoDrop 1000 Spectrophotometer or electrophoresis on 1 % agarose gel. Genomic DNA was removed from the total RNA using Rnase-free DNase before cDNA synthesis. All cDNA samples were synthesized from 1 mg total RNA with the PrimeScript RT Reagent Kit. The first-strand cDNA was synthesized by the PrimeScript RT Enzyme following the manufacturer’s instructions with random primers.

### cDNA cloning of *UCHL1*

A partial cDNA sequence of *UCHL1* was acquired from the transcriptome database for the Chinese giant salamander in our lab. The primer for amplifying UCHL1 cDNAs fragments were designed (UCHL1-F and UCHL1-R) and is shown in Table 1. PCR amplification was performed as follows: 1 cycle of 94°C for 5 min, 35 cycles, including denaturation, at 94°C for 40 s, annealing at 56°C for 40 s, and extension at 72°C for 1 min, followed by 1 cycle at 72°C for 10 min. The specific products were assessed by electrophoresis on 1 % agarose gel and cloned into the plasmid vector pMD19-T and then transformed into *E. coli* TOP10 for sequencing. The nucleotide sequences were determined for three sense and antisense strands of plasmid inserts for three independent clones.

**Table 1:** Primer pairs in the study

Primer	Sequence (5′ to 3′)	Nucleotide position (nt)	Remark
UCHL1-F	CCAAAGAGCACGCAGACATGC	179-299	UCHL1 fragment
UCHL1-R	TTAAGACTTGCTGAGCGCCAC	844-864	
AP	GGCCACGCGTCGACTAGTACT18		3′ Race
AAP	GGCCACGCGTCGACTACGGGIIGGGIIGGGIIG		5′Race
AUAP	GGCCACGCGTCGACTAGTAC		3′, 5′Race
5′RTP	GGCATTCGTCCATCCAGCTC	715-734	5′Race
5′GSP1	GGCATTCGTCCATCCAGCTC	715-734	5′Race
5′GSP2	GTTCACTTTGTCATCCACCC	653-672	5′Race
5′GSP3	CTTTGTCATCCACCCGGCAG	658-677	5′Race
3′GSP1	CCCTCTCACTCCTCAGCATG	354-373	3′Race
3′GSP2	CTGTGCTGTTCTTCTGCTCT	333-352	3′Race
3′GSP3	GGATGTGCTTGAGTTCTCAG	282-302	3′Race
F-UCHL1	GTGGTTAGAGGTCCAAAGAG	167-186	qPCR
R-UCHL1	ATCCACGAAGTGCCAACCAG	266-285	
F-β-actin	AAAAGCCAACCGAGAAAAG		qPCR
R-β-actin	TACGACCAGAGGCATACAG		

To obtain the 5′ and 3′ ends of each cDNA, a number of gene-specific sense or antisense primers (Table 1) were designed for the three UCHL1 transcripts to replicate sequence regions for their core fragment sequenced above. 5′- and 3′-RACE were performed with the RACE cDNA Amplification Kit using ovarian or liver total RNA according to manufacturer’s protocols, respectively. Nested 5′- and 3′-RACE products of the expected size were sub-cloned and sequenced as described above.

### Bioinformatic analysis of UCHL1

The complete ORF regions and amino acid sequences of UCHL1 were deduced using ORFfinder at NCBI (http://www.ncbi.nlm.nih.gov/gorf/gorf.html). The cleavage site for the signal peptide was predicted with SignalP (http://www.cbs.dtu.dk/services/SignalP/). O-linked and N-linked glycosylation sites were detected with the NetOGlyc Server (http://www.cbs.dtu.dk/services/NetOGlyc/) and with the NetNGlyc Server (http://www.cbs.dtu.dk/services/NetNGlyc/), respectively. To evaluate the evolutionary relationship of CGS UCHL1 with 13 other species, nucleic acid and amino acid sequences of the UCHL1 protein from different species were used to perform multiple alignments using ClustalX2 and a phylogenic tree was constructed by the neighbor-joining (NJ) method within MEGA version 5.0 [30]. GenBank accession numbers of the UCHL1 amino acid sequences included in the tree are: BC005117.1 (*H. sapiens*), NM_011670.2 (*M. musculus*), NM_001080212.1 (*G. gallus*), XM_019553393.1 (*C. porosus*), AY147851.1 (*D. rerio*), NM_001096657.2 (*X. laevis*), XM_007069383.1 (*C. mydas*), AB490372.1 (*L. reevesii* rubritaeniata), NM_057592.4 (*D. melanogaster*).

### Developmental expression of UCHL1 mRNA in testes of CGSs at different stages

The expression patterns of UCHL1 in different developmental stages of gonads were detected by RT-qPCR. The qRT-PCR mixture consisted of 1 μL of the diluted cDNA samples (50 ng/ μL), 6 μL Power 2×SYBR Real-time PCR premixture (BioTeKe), 1 μL of each primer (0.4 μM), and 11 μL H_2_O. The qRT-PCR cycle profile included 1 cycle at 95°C for 5 min, then 32 cycles at 95°C for 15 s, 58°C for 15 s, and 72°C for 20 s. To determine the relative mRNA expression level of *UCHL1*, QRT-PCR was performed via the 2-ΔΔ^ct^ method with the *β-actin* gene used as an internal control gene for cDNA normalization. The special primer pairs for *UCHL1* (F-UCHL1, R-UCHL1) and *β-actin* (F-β-actin, R-β-actin) were designed and are listed in Table 1.

### Histological analysis

For histological analysis, tissues from the normal CGSs were dissected and fixed in Bouin’s fixative overnight. After successive dehydration, tissues were imbedded in paraffin. Sections (5 μm) were mounted on slides with polylysine pretreatment, dewaxed in a xylene bath, and then rehydrated through gradient ethanol solutions. The sections were used for immunohistochemistry (IHC). IHC was performed for locating the UCHL1 in the tissues. After rinsing in PBS, heat-mediated antigen retrieval was performed in boiling citrate for 10 min. Slides were cooled for 40 min at room temperature and then rinsed three times with PBS before treating with 3 % H_2_O_2_. Nonspecific protein binding was blocked for 1 h by normal goat serum before incubating with the UCHL1 antibodies (1:200; Cat: ab916, Abcam) overnight in a damp box. Slides were washed (3 × 5 min) in PBS, followed by incubation for 1 h with biotinylated anti-mouse. Next biotinylated horseradish peroxidase was used for 1 h. Slides were stained by DAB according to the manufacturer’s instructions before counterstaining with hematoxylin. Images were obtained using a microscope. As controls, the primary antibodies (rabbit anti-UCHL1, Millipore, 1:200) were replaced with PBS.

### Testes sample collection and protein extraction

The testes were harvested at different developmental stages. Firstly, the testes were split using small forceps on ice and 10 mg tissue fragments were washed using 200 μL PBS containing 1 % protease inhibitors. Subsequently, the samples were centrifuged at 800 *g* for 10 min at 4°C. The pellets and the supernatant fractions (tissue liquid) were collected for protein extraction. Thirdly, the pellets were lysed using RIPA buffer containing 1 % protease inhibitors. The lysates and the tissue liquid were cleared by centrifugation at 14,000 *g* for 20 min. Then the concentration of protein was analyzed using the BCA method.

### Separation and identification of exosomes from the adult testes

#### Separation of exosomes

The extracellular vesicles (or exosomes) was separated from the adult testes. Firstly, the testes were split by small forceps on ice and 10 mg tissue fragments were washed using 200 μL PBS containing 1 % protease inhibitors. Subsequently, the samples were centrifuged at 800 *g* for 10 min at 4°C. The supernatant was centrifuged at 12,000 *g* for 20 min and the supernatant was filtered through 0.22-μm membranes. Subsequently, ultracentrifugation at 120,000 *g* for 2 h was performed to collect exosomes. Finally, exosomes pellets were resuspended in 200 μL cold PBS.

#### Transmission electron microscopy (TEM)

Fresh isolated exosomes were diluted in cold PBS through serial dilutions. After mounting on copper grids, the samples were fixed in 1 % glutaraldehyde for 10 min, washed in sterile distilled water, and then incubated with phosphotungstic acid for 1–2 min. Excess reagent was removed and samples were dried at room temperature. Finally, the HT7700 TEM was used to image the exosome samples at 80–100 kV.

#### Scanning electron microscopy (SEM)

The method was as previously described, with minor modifications [31]. Exosomes were fixed in 2.5 % glutaraldehyde for 1 h at 4°C. The samples were diluted in distilled water through serial dilutions, and then 5 μL were added to a cleaned silicon slice. After air drying under a ventilation hood, exosomes were post-fixed in 1 % osmic acid for 1 h and then dehydrated in increasing concentrations of ethyl alcohol (35, 50, 70, 80, 90, and 100 %) every 20 min. Next, isoamyl acetate was used to treat samples for 10 min. Finally, the specimens were sputter-coated with gold palladium and viewed under a scanning electron microscope.

#### Nanoparticle tracking analysis

Size distribution and quantification of the extracellular vesicles were analyzed using a NanoSight NS300 instrument as described previously [32]. Briefly, exosomes from testes were diluted in 1mL of PBS and disaggregated by using a syringe and needle. Then, the sample was injected into the chamber and three individual samples were tested.

### Western blot analysis

Protein samples were boiled in 5× loading buffer for 10 min, and separated by sodium dodecyl sulfate-polyacrylamide gel electrophoresis. Subsequently, proteins were transferred to a polyvinylidene difluoride membrane. Then, the membranes were blocked with 5 % (w/v) skim milk and 0.05 % (v/v) Tween 20 in Tris buffered saline (TBS; 20 mmol/L Tris, 500 mmol/L NaCl; pH 7.0). The mouse anti-UCHL1 (1:200, Cat:ab916, Abcam) was used as the primary antibodies and incubated with the membrane at a dilution of 1:500 overnight at 4°C. The membranes were washed with TBS containing 0.05 % (v/v) Tween 20 and subsequently incubated with HRP-conjugated anti-mouse IgG antibody at a dilution of 1:1000. Finally, after the membrane was incubated with ECL for chemiluminescence developing by BIO-RAD ChemiDoc XRS. β-actin antibody was used to detect β-actin protein as an internal control in the examination.

### Statistical analyses

All experiments were performed with at least three replicates. Data were expressed as mean ± standard deviation (S.D.) and were analyzed with an analyses of variance (ANOVA) and Duncan’s multiple comparisons. The statistical analyses were conducted with SPSS software. The significance level was defined as *p* < 0.05.

## Abbreviations

UCHL1: Ubiquitin carboxyl-terminal hydrolase L1
CDS: Coding sequences
CGS: Chinese giant salamander
ORF: Open reader form
IHC: Immunohistochemistry
PBS: Phosphate buffer saline
DAB: Diaminobenzidine
TEM: Transmission electron microscopy
SEM: Scanning electron microscopy
TBS: Tris buffered saline
